# Overexpression of Long Non-Coding RNA Linc01315 Predicts Poor Prognosis in Breast Cancer

**DOI:** 10.3389/fonc.2021.562378

**Published:** 2021-10-05

**Authors:** Jingyan Xue, Sheng Huang, Jiaying Chen, Yi-zuo Chen, Zhi-min Shao, Jiong Wu, Yayun Chi

**Affiliations:** ^1^ Key Laboratory of Breast Cancer in Shanghai, Department of Breast Surgery, Fudan University Shanghai Cancer Center, Shanghai, China; ^2^ Department of Oncology, Shanghai Medical College, Fudan University, Shanghai, China; ^3^ The 2nd Department of Breast Surgery, Breast Cancer Center of the Third Affiliated Hospital of Kunming Medical University, Tumor Hospital of Yunnan Province, Kunming, China; ^4^ Department of Head and Neck Surgery, Fudan University Shanghai Cancer Center, Shanghai, China; ^5^ Department of Thyroid and Breast Surgery, The First Affiliated Hospital of Wenzhou Medical University, Wenzhou, China; ^6^ Collaborative Innovation Center for Cancer Medicine, Shanghai Medical College, Fudan University, Shanghai, China

**Keywords:** Linc01315, breast cancer, prognosis, long non-coding RNA, predict

## Abstract

**Background:**

LncRNAs have been shown to play critical roles in regulating tumorigenesis and tumor progression. Using LncRNAs to predict prognosis and therapeutic response to cancer treatment has been caused for concern, but the predictive value of lncRNAs remains to be explored and underlying mechanisms have not been completely understood.

**Methods:**

The Linc01315 expression level was detected in 282 breast cancer tissues by using quantitative RT-PCR. The association between Linc01315 expression level and clinicopathological features of these breast cancer patients was further analyzed. Multiple regression analysis was used to evaluate Linc01315 predictive value of patients’ prognosis.

**Results:**

Our study revealed that Linc01315 expression level was significantly correlated with vessel invasion (*P* = 0.028) and tumor subtype (*P* = 0.039). The Kaplan–Meier survival curves demonstrated that patients with lower Linc01315 expression level had significantly longer disease free survival (DFS) (*P* = 0.002) and overall survival (OS) (*P*=0.019). Multiple regression analysis showed that Linc01315 level could be an independent predictive factor for DFS (hazards ratio = 0.613, 95% confidence interval = 0.375-1.003; *P* = 0.049) and OS (hazards ratio = 0.439, 95% confidence interval = 0.228-0.845; *P* = 0.014). Further analysis showed that low Linc01315 level patients with endocrine therapy could benefit patients DFS (*P*=0.037) and OS (*P*=0.025).

**Conclusion:**

Our results demonstrate that Linc01315 expression level is significantly correlated with breast cancer patients’ prognosis. Linc01315 may represent an independent prognostic marker and therapeutic target in breast cancer.

## Introduction

Breast cancer is the most common malignancy in women. The overall survival and prognosis of some patients with breast cancer remains poor despite therapeutic improvements in cancer systemic treatment over the last several decades. Thus, there is still a great challenge in exploring the underlying mechanisms of breast carcinogenesis and progression, so as to identify promising prognostic molecular markers that can benefit the effect of appropriate therapeutic regimen.

Emerging evidence has indicated that long non-coding RNA (lncRNA) could be involved in the occurrence and progression of cancer. Long non-coding RNAs (lncRNAs), a subgroup of non-protein-coding RNAs, are longer than 200 nucleotide (1, 2) and generally show developmental stage-, tissue-, or disease-specific expression patterns (3–7). Ectopic lncRNA expression levels have been implicated in various types of cancer development and metastasis through the downstream effector molecules dysregulation. lncRNA expression profiling may act as prognostic markers and promising therapeutic targets for human cancers intervention (8–12). Although thousands of lncRNAs have been annotated, only a small number of lncRNAs, such as homeobox transcript antisense RNA (HOTAIR) and metastasis-associated lung adenocarcinoma transcript 1 (MALAT1), are widely studied in various tumors (13–16); however, the functions of most lncRNAs remain unknown.

Previous studies have shown that TCGA lncRNome information could be used to screen and find several lncRNA candidates that associated with cancer genomic alterations, and might be correlated with patient prognosis in breast cancer (17, 18). Linc01315 located on chromosome 12 which has been reported to be upregulated in triple-negative breast cancer (19). Moreover, down-regulated expression of Linc01315 could suppress colorectal carcinoma progression by sponging miR-205-3p (20). In this study, we investigated Linc01315 expression level in the cohort of 282 breast cancer patients from Fudan University Shanghai Cancer Center and found that high Linc01315 expression level had a significantly worse clinical outcome, and Linc01315 might be an independent prognostic predictor in breast cancer.

## Material and Methods

### Cell Culture

The MCF-10A, MCF-7,SK-BR-3,T47D and BT474 cell lines were obtained from American Type Culture Collection (ATCC) (Manassas, VA). MDA-MB-231/LM2 was a subline of MDA-MB-231 with high lung metastasis tendency. MCF-7,SK-BR-3, T47D and BT474 cells were cultured in RMPI1640 medium. MDA-MB-231/LM2 cell was cultured in DMEM medium. MCF-10A was cultured in F12/DMEM 1:1 medium. All cells were cultured with 10% fetal bovine serum(FBS), 100 units/ml penicillin, and 100 ug/ml streptomycin at 37°C and 5% CO_2_.

### Patients’ Samples

A series of 282 primary breast cancer tissues of stage I to III invasive carcinoma were collected at Fudan University Shanghai Cancer Center. The exclusion criteria were: (i) advanced breast cancer, (ii) neo-adjuvant chemotherapy received prior to surgery, (iii) recurrent breast cancer diagnosed upon surgery, and (iv) noninvasive ductal carcinoma. Patients’ clinical data and tumor pathology are summarized (**Table 1**). Pathology, including histological type and grade; ER, PR, and HER2 status; and Ki-67 expression were assessed by two academic pathologists according to the World Health Organization (WHO) classification and American Society for Clinical Oncology (ASCO) guidelines. The study was approved by the Ethical Committee of Fudan University Shanghai Cancer Center for Clinical Research (NO. 050432-4-1911D). All patients gave written informed consents.

**Table 1:** Clinical information and demographics of the 282 patients included in the study.

Characteristics	LINC01315		Number of patients (%)	p value
	Low N (%)	High N (%)		
**Total**	167 (59.2)	115 (40.8)	282	
**Age**				0.903
<50	74 (26.2)	52 (18.5)	126 (44.7)	
≥50	93 (33.0)	63 (22.3)	156 (55.3)	
**Tumor size (cm)**				0.061
≤2 cm	60 (21.3)	43 (15.2)	103 (36.5)	
>2, ≤5 cm	105 (37.2)	65 (23.1)	170 (60.3)	
>5 cm	2 (0.7)	7 (2.5)	9 (3.2)	
**Node status**				0.15
N0	89 (31.6)	50 (17.7)	139 (49.3)	
N1	43 (15.2)	27 (9.6)	70 (24.8)	
N2	15 (5.3)	16 (5.7)	31 (11.0)	
N3	20 (7.1)	22 (7.8)	42 (14.9)	
**Tumor Grade**				0.134
DCIS-IDC I	10 (3.5)	5 (1.8)	15 (5.3)	
IDC II	85 (30.1)	47 (16.7)	132 (46.8)	
IDC III	69 (24.5)	66 (23.4)	135 (47.9)	
**TNM Stage**				0.003*
I-II	133 (47.2)	74 (26.2)	207 (73.4)	
III	31 (11.0)	44 (15.6)	75 (26.6)	
**ER status**				0.181
Negative	70 (24.8)	58 (20.6)	128 (45.4)	
Positive	97 (34.4)	57 (20.2)	154 (54.6)	
**PR status**				0.274
Negative	72 (25.5)	58 (20.6)	130 (46.1)	
Positive	95 (33.7)	57 (20.2)	152 (53.9)	
**Her-2 status**				0.376
Negative	112 (39.7)	71 (25.2)	183 (64.9)	
Positive	55 (19.5)	44 (15.6)	99 (35.1)	
**Ki67**				0.395
Negative	79 (28.0)	48 (17.0)	127 (45.0)	
Positive	88 (31.2)	67 (23.8)	155 (55.0)	
**Vessel invasion**				0.028*
Negative	100 (35.5)	53 (18.8)	153 (54.3)	
Positive	67 (23.7)	62 (22.0)	129 (45.7)	
**Subtype**				0.039*
Luminal A	51 (18.1)	31 (11.0)	82 (29.1)	
Luminal B	48 (17.0)	26 (9.2)	74 (26.2)	
Her-2	28 (9.9)	36 (12.8)	64 (22.7)	
Basal like	40 (14.2)	22 (7.8)	62 (22.0)	

Fisher’s exact test was used to analyze categorical variables. *P < 0.05.

ER, estrogen receptor; PR, progesterone receptor; Her2, human epidermal growth factor receptor 2; DCIS, ductal carcinoma in situ; IDC, invasive ductal carcinoma.

### RNA Isolation and Quantitative RT−PCR

Total RNA was extracted with Trizol reagent (Invitrogen, Carlsbad, CA, USA) according to the manufacturer’s instructions. DNase I was used to digest genome DNA before cDNA Synthesis. qRT-PCR was performed to quantitate the mRNA expression levels. To analyze Linc01315 expression, we used the SYBRGreen method with primers listed below: Linc01315 forward 5′ CTGCTGAGCGATGAAGTGGA 3′ and Linc01315 reverse 5′ CTACAGCTGGAGGGAAACCG 3′. Their relative expression were quantified to GAPDH by 2^−△Ct^ values.

### Statistical Analysis

All statistical analyses were performed using SPSS 22.0 software (SPSS Inc, Chicago, IL, USA). Comparisons among groups were done by the independent sample t-test. The Wilcoxon matched pairs test was used to examine Linc01315 expression in breast cancer tissues *versus* adjuvant normal tissues. Pearson’s χ2 test was performed to evaluate the correlation between Linc01315 expression and breast cancer clinicopathological characteristics. In survival analysis, Kaplan–Meier analysis and the Cox proportional hazards model were used to examine whether Linc01315 expression level impacted prognosis. Two-tailed P-values were adopted, P-value 0.05 was considered as statistically significant. We used SPSS 22.0 software (SPSS Inc, Chicago, IL, USA) to draw survival curve.

## Results

### Linc01315 Expression in Breast Cancer Cell Lines

**Figure 1:**
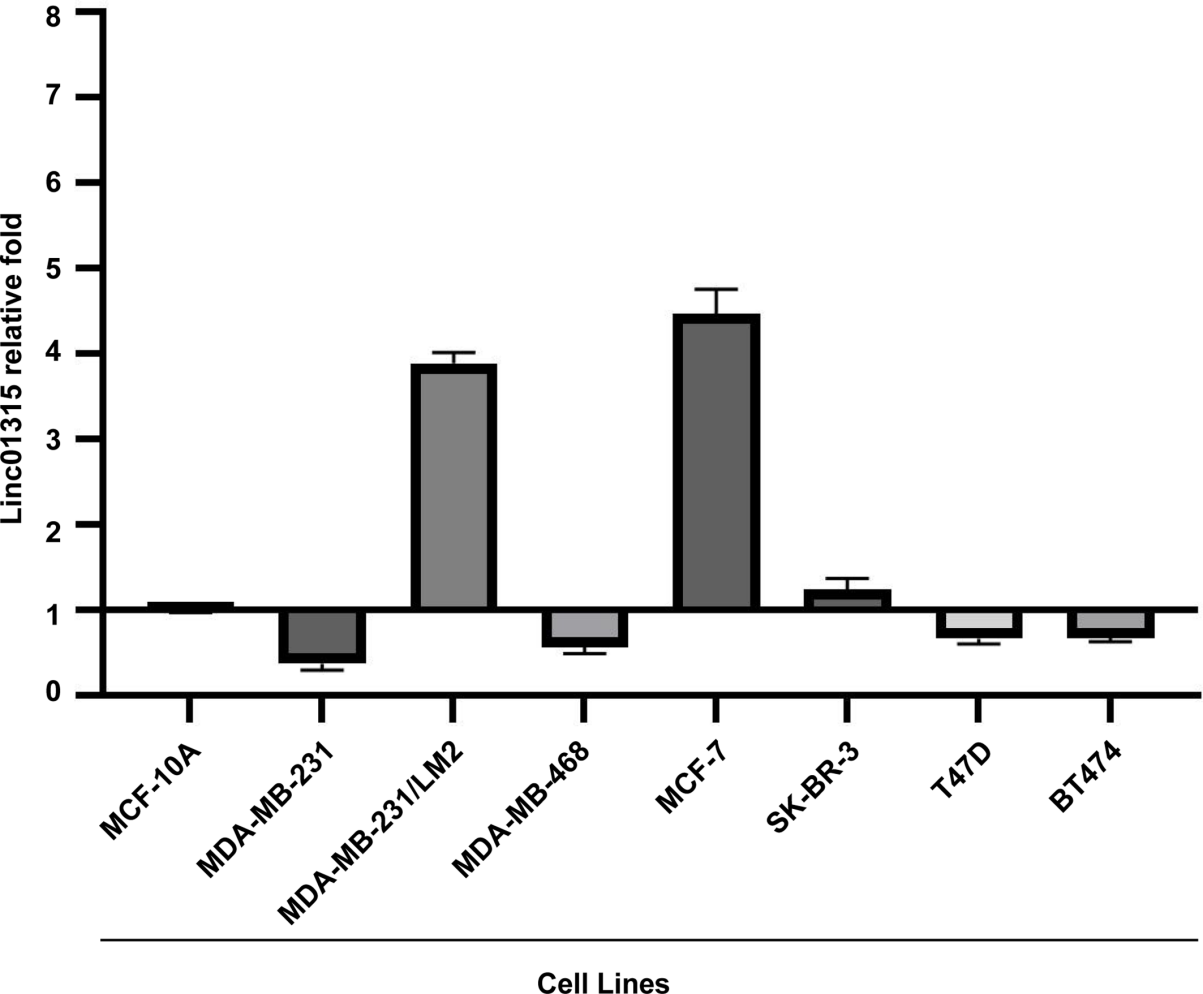
Linc01315 expression levels in 6 breast cell lines. Compared with normal breast cell line MCF-10A. Linc01315 expression was up-regulated in MDA-MB-231/LM2, MCF-7 and SK-BR-3, down-regulated in T47D and BT474.

Linc01315 expression levels were examined in seven breast cancer cell lines and one normal breast cell MCF10A. As shown in **Figure 1**, compared with MCF-10A. Linc01315 expression was up-regulated in MDA-MB-231/LM2, MCF-7 and SK-BR-3, however, down-regulated in MDA-MB-231, MDA-MB-468, T47D and BT474.

### Relationship Between Linc01315 Expression Level and Clinicopathological Characteristics

Expression of Linc01315 in 44 paired of breast cancer tissues and their adjacent normal tissues were then detected. The data showed that a little bit higher of Linc01315 was in normal tissues than in breast cancer tissues, but the difference was not significant (P=0.1038) (**Supplementary Figure 1**). To identify the clinical relevance of Linc01315 in breast cancer, association between Linc01315 expression level and clinicopathological features such as age, ER/PR, Her-2, Ki67, vessel invasion, tumor size, lymph node status, tumor grade and TNM stage were examined (**Table 1**). According to the ROC curve assessment which was constructed using patient Linc01315 levels to establish a sensitivity-specificity relationship of the measured prognostic marker, the expression of Linc01315 in tumor tissues was divided into 167 low expression and 115 high expression. Chi square test was used to determine the cutoff value of Linc01315 in this study. Linc01315 expression level in breast cancer was significantly correlated with TNM stage (*p*=0.003), vessel invasion (*p* = 0.028) and tumor subtype (*p* = 0.039). TNM stage I-II patients seemed to have lower Linc01315 expression. Vessel invasion negative patients show significant correlation with low Linc01315 expression level. Moreover, Her-2 positive subtype patients tended to have high Linc01315 expression level, while luminal A and B subtype patients seemed to have low Linc01315 expression (**Table 1**). The expression levels of Linc01315 were higher in Luminal A and Her-2 subtype than in Luminal B and basal like subtype (**Supplementary Figure 2**).

### Association Between Linc01315 Expression and Patient Prognosis

**Figure 2:**
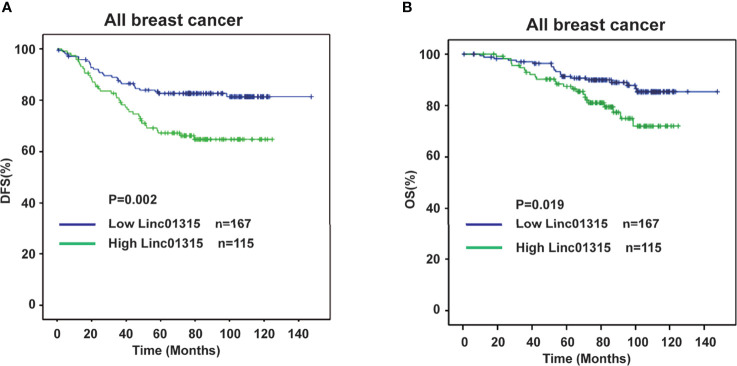
Low expression level of Linc01315 correlates with higher disease free survival (DFS) and overall survival (OS). **(A)** Kaplan-Merier curves of 282 breast cancer patients after stratification by the level of Linc01315 were used for depicting Disease-free survival (DFS). **(B)** Kaplan-Merier curves of 282 breast cancer patients after stratification by the level of Linc01315 were used for depicting Overall survival (OS).

Furthermore, analyzing the disease free survival (DFS) and overall survival (OS) of these 282 breast cancer patients revealed that patients with lower Linc01315 expression level had significantly longer DFS (*P* = 0.002) and OS (*P*=0.019) (**Figures 2A, B**). Interestingly, the tumor stage, lymph node stage, TNM stage and vessel invasion also showed significant correlation with patients DFS and OS, as patients with lower tumor stage (*P* = 0.036, *P*=0.046), lower lymph node stage (*P*<0.001, *P*=0.041), lower TNM stage (*P*<0.001, *P*=0.003) or negative vessel invasion (*P*<0.001, *P*=0.002) tended to achieve longer DFS and OS. Furthermore, Her-2 positive patients displayed worse OS (*P*=0.042). Multiple regression analysis showed that the Linc01315 level could serve as an independent predictive factor for DFS (hazards ratio = 0.613, 95% confidence interval = 0.375-1.003; *P* = 0.049) and OS (hazards ratio = 0.439, 95% confidence interval = 0.228-0.845; *P* = 0.014) (**Table 2**). Statistical analysis also showed that lymph node stage and TNM stage could predict patients DFS (hazards ratio = 1.644, 95% confidence interval = 1.344-2.010; *P*<0.001) (hazards ratio = 2.413, 95% confidence interval = 1.648-3.531; *P*<0.001) and vessel invasion status could predict patients OS (hazards ratio = 2.590, 95% confidence interval = 1.303-5.149; *P* = 0.007) (**Table 2**).

**Table 2:** Univariate and multivariate Cox regression analyses of DFS and OS in BC patients.

Covariates	Univariate analysis		Multivariate analysis	
	HR (95% CI)	*P*-value	HR (95% CI)	*P*-value
***DFS***				
**Age (≤50 *versus*>50 years)**	0.673 (0.418-1.084)	0.103	**—**	
**T stage (T2/3 *versus* T1)**	1.780 (1.039-3.052)	0.036*	1.115 (0.700-1.775)	0.647
**pN stage**		<0.001*	1.644 (1.344-2.010)	<0.001*
**N1 *versus* N0**	0.196 (0.103-0.375)	<0.001*		
**N2 *versus* N0**	0.422 (0.223-0.798)	0.008*		
**N3 *versus* N0** **Tumor Grade (DCIS/IDC I-II *versus* IDC III)** **TNM Stage (I-II *versus* III)**	0.781 (0.388-1.569) 1.108 (0.786-1.564) 2.626 (1.799-3.832)	0.487 0.558 <0.001*	**—** 2.413 (1.648-3.531)	<0.001*
**ER (positive *versus* negative)**	1.321 (0.809-2.155)	0.266	**—**	
**PR (positive *versus* negative)**	1.379 (0.845-2.250)	0.194	**—**	
**HER2 (positive *versus* negative)**	0.668 (0.393-1.135)	0.136	**—**	
**Vessel invasion (positive *versus* negative)**	2.655 (1.606-4.390)	<0.001*	1.249 (0.641-2.434)	0.514
**LINC01315 expression level (high *versus* low)**	2.051 (1.267-3.320)	0.003*	0.613 (0.375-1.003)	0.049*
***OS***				
**Age (≤50 *versus* >50 years)**	0.724 (0.386-1.357)	0.313	**—**	
**T stage(T2/3 *versus* T1)**	2.136 (1.014-4.501)	0.046*	1.541(0.894-2.657)	0.120
**pN stage**		0.041*	1.028(0.735-1.438)	0.871
**N1 *versus* N0**	0.379 (0.160-0.899)	0.028*		
**N2 *versus* N0**	0.648 (0.263-1.594)	0.345		
**N3 *versus* N0** **Tumor Grade (DCIS/IDC I-II *versus* IDC III)** **TNM Stage (I-II *versus* III)**	1.230 (0.474-3.190) 1.342 (0.824-2.187) 2.095 (1.296-3.384)	0.670 0.237 0.003*	**—** 1.397 (0.787-2.481)	0.254
**ER (positive *versus* negative)**	1.008 (0.528-1.924)	0.980	**—**	
**PR (positive *versus* negative)**	1.070 (0.561-2.039)	0.838	**—**	
**HER2 (positive *versus* negative)**	0.446 (0.205-0.969)	0.042*	0.442 (0.203-0.964)	0.04*
**Vessel invasion (positive *versus* negative)**	2.982 (1.510-5.890)	0.002*	2.590 (1.303-5.149)	0.007*
**LINC01315 expression level (high *versus* low)**	1.893 (1.007-3.557)	0.048*	0.439 (0.228-0.845)	0.014*

CI, confidence interval; HR, hazard ratio; DFS,disease-free survival;OS, overall survival; BC, breast cancer. *P < 0.05.

**Figure 3:**
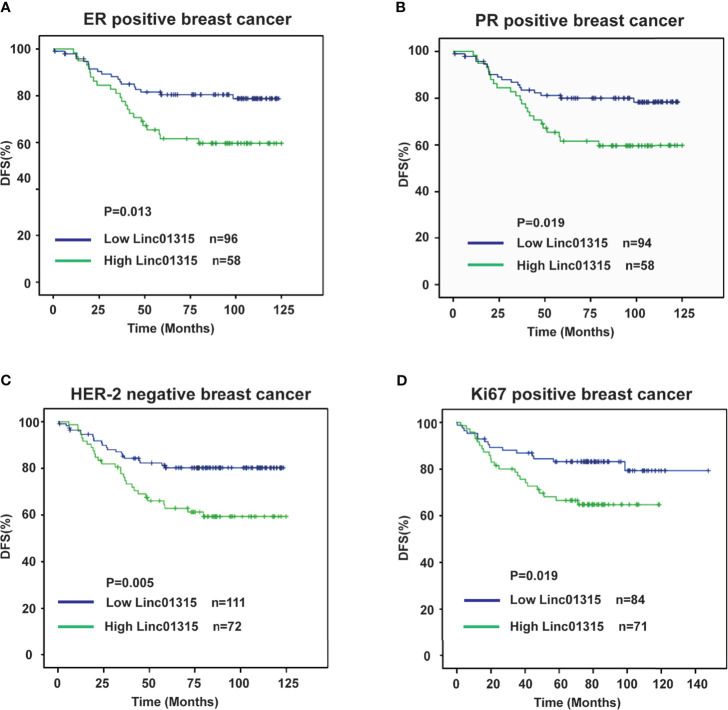
Disease-free survival curves according to breast cancer patients’ clinic pathological features and Linc01315 expression level. **(A)** DFS curve of ER positive patients based on the level of Linc01315. **(B)** DFS curve of PR positive patients based on the level of Linc01315. **(C)** DFS curve of Her-2 negative patients based on the level of Linc01315. **(D)** DFS curve of Ki67 positive patients based on the level of Linc01315.

**Figure 4:**
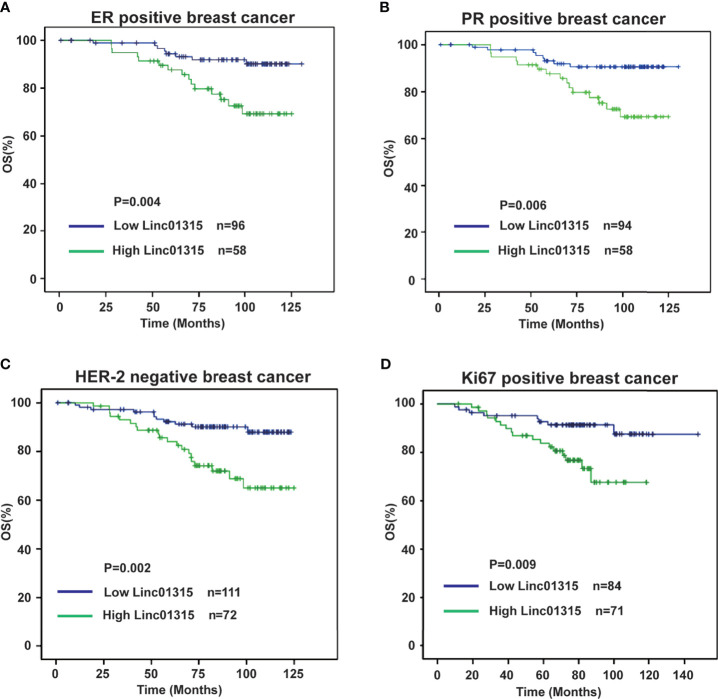
Overall survival curves according to breast cancer patients’ clinic pathological features and Linc01315 expression level. **(A)** OS curve of ER positive patients based on the level of Linc01315. **(B)** OS curve of PR positive patients based on the level of Linc01315. **(C)** OS curve of Her-2 negative patients based on the level of Linc01315. **(D)** OS curve of Ki67 positive patients based on the level of Linc01315.

**Figure 5:**
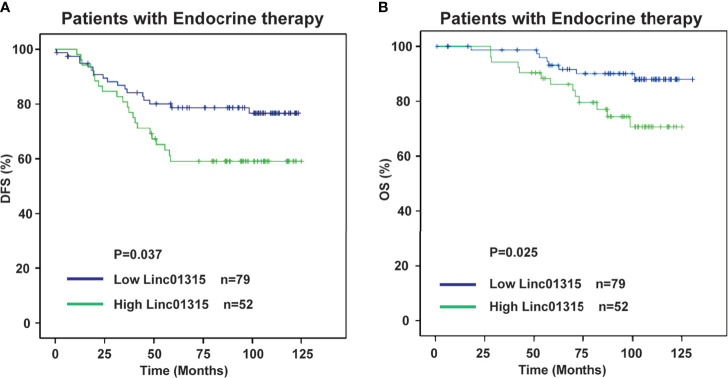
Low Linc01315 level patients with endocrine therapy could benefit patients DFS and OS. **(A)** DFS curve of patients treated with endocrine therapy based on the level of Linc01315. **(B)** OS curve of patients treated with endocrine therapy based on the level of Linc01315.

Further analysis showed that the ER/PR positive patients with lower Linc01315 expression displayed longer DFS (*P*=0.013; *P*=0.019) and OS (*P*=0.004; *P*=0.006) (**Figures 3A, B**; **Figures 4A, B**), while OS and DFS analysis in HER2+ breast cancer and triple negative breast cancer showed no significant difference (**Supplementary Figure 3**). ER positive breast cancer patients receive endocrine therapy to block estrogen action in the tumor, consequently stop or slow the development of hormone-sensitive tumors. Additionally, we investigated the prognosis of patients treated with endocrine therapy (n = 131). The 131 ER positive patients received Tamoxifen or Aromatase inhibitor for 5 years based on menopausal status at diagnosis. We found low Linc01315 level patients with endocrine therapy could benefit patients DFS (*P*=0.037) and OS (*P*=0.025) (**Figures 5A, B**).

Nevertheless, Her-2 negative patients with high Linc01315 expression showed poor DFS (*P*=0.005) and OS (*P*=0.002) (**Figures 3C**, **4C**). Ki67 gene encodes a nuclear protein that is associated with and may be necessary for cellular proliferation. Ki67 positive patients with lower Linc01315 expression seemed to benefit DFS (*P*=0.019) and OS (*P*=0.009) (**Figures 3D**, **4D**).

## Discussion

Increasing efforts to achieve effective personalized treatment have emphasized the importance of applicable molecular markers to predict patient prognosis and therapeutic effect.

Multiple studies have indicated that LncRNAs can function as tumor suppressors or oncogenes through regulating coding genes expression and epigenetic modification (21). Molecular mechanisms of lncRNAs in various cancers may represent the key points for therapeutic intervention. Linc01315 is located in chromosome 22q. LINC01315 has three variants, NR_120595, NR_120596, and NR_120597, and the primers we designed could recognize these three isoforms. A previous study speculated that Linc01315 could promote the proliferation, migration and invasion of nasopharyngeal carcinoma (NPC) cells, and reduced the apoptosis of NPC cells, resulting in facilitating the tumorigenesis of NPC (22). The study demonstrated that Linc01315 might be used for the early diagnosis and treatment of NPC. In our study, we identified that Linc01315 expression level might be associated with breast cancer patients’ prognosis. High Linc01315 expression in breast cancer significantly correlated with worse DFS and OS (**Figures 2A, B**). Multiple regression analysis displayed that the Linc01315 level could be an independent predictive factor for DFS and OS in breast cancer (**Table 2**).

To our knowledge, about 70% of breast cancer patients presenting ER/PR positive (23). ER mediates the effects of estrogen on the development and progression of breast cancer, and it acts as an important therapeutic target for treatment. Endocrine therapy resistance in breast cancer is a major challenge in the treatment of hormone receptor positive breast cancer. Several lncRNAs have been reported to play crucial roles in the modulation of endocrine therapy responses in breast cancer cells. Previous study showed that lncRNA UCA1 could confer tamoxifen resistance by activating the mTOR signaling pathway (24) and Wnt/β-Catenin signaling pathway (25). LncRNA HOTAIR was found to interact with ER, and then enhance its transcriptional activity and promote tamoxifen-resistant breast cancer progression (26). Our study showed that Linc01315 expression level in breast cancer was significantly correlated with tumor subtype, the expression level of Linc01315 was higher in Luminal A and Her-2 subtype than in Luminal B and basal like subtype (**Table 1** and **Supplementary Figure 2**). However, previous study showed that the expression level of Linc01315 was higher in triple negative breast cancer(TNBC) tissues compared with non-TNBC tissues, and high Linc01315 expression associated with better DFS in TNBC patients (19). Considering samples selection bias, we may need to expand the sample size, and design a prospective trial in the further study. Further analysis also revealed that ER/PR positive patients with lower Linc01315 expression displayed longer DFS and OS (**Figures 3A, B**; **Figures 4A, B**), and low Linc01315 level patients treated with endocrine therapy could benefit patients DFS and OS (**Figures 5A, B**). The results indicate that Linc01315 may be associated with endocrine therapy resistance in breast cancer, and represent a promising therapeutic target for cancer treatment.

In the past decade, lncRNA research is booming. Therapeutics based on targeting lncRNAs has evolving rapidly. It has been well demonstrated that multiple RNA-based therapies, such as small interfering RNAs (siRNAs), short hairpin RNAs (shRNAs), antisense oligonucleotides (ASOs), as well as CRISPR-Cas13a based RNA editing, showed promising results in preclinical research. In the ASOs scenario, different chemical modifications have been developed to enhance the stability of this kind of drug, also the delivery efficiency to tumor cells. ASOs are single stranded chemically modified DNA/RNA synthetic molecules complementary to target mRNA, causing mRNA degradation through RNase H cleavage or stabilizing the targets. We have demonstrated the efficiency of ASOs in the treatment of triple-negative breast cancer cells targeting LINC02273 *in vivo (* 12). Therefore, our study could also utilize this tool for future translational study although it may need more mechanism and animal studies.

In conclusion, our data demonstrate that Linc01315 expression level is significantly correlated with breast cancer patients’ prognosis. Lower Linc01315 expression level may benefit patients DFS and OS, especially in ER/PR positive patients treated with endocrine therapy. Linc01315 also has prognostic effect in Her-2 negative or Ki67 positive breast cancer patients. It is plausible that Linc01315 may represent an independent prognostic marker and therapeutic target in breast cancer.

## Data Availability Statement

The raw data supporting the conclusions of this article will be made available by the authors, without undue reservation.

## Ethics Statement

This study was approved by the Ethical Committee of Fudan University Shanghai Cancer Center for Clinical Research. Written informed consents were obtained from all the patients.

## Author Contributions

YaC and JW designed the research study. JX, SH, and Y-zC conducted the experiments. JX, SH and JC collected clinical data. JX, JC and Z-mS analyzed the data. JX wrote the manuscript with contribution from all authors. All authors contributed to the article and approved the submitted version.

## Funding

This work was supported by the National Key R&D Program of China (2017YFC1311004) and National Natural Scientific Foundation of China (81874115 to YaC, 81602324 to JX, and 81772815 to JW).

## Conflict of Interest

The authors declare that the research was conducted in the absence of any commercial or financial relationships that could be construed as a potential conflict of interest.

## Publisher’s Note

All claims expressed in this article are solely those of the authors and do not necessarily represent those of their affiliated organizations, or those of the publisher, the editors and the reviewers. Any product that may be evaluated in this article, or claim that may be made by its manufacturer, is not guaranteed or endorsed by the publisher.
