# Supplementary figures and images for: Overexpression of Long Non-Coding RNA Linc01315 Predicts Poor Prognosis in Breast Cancer
Source: Front Oncol. 2021 Oct 5;11:562378. doi: 10.3389/fonc.2021.562378 (PMC8524084; doi:10.3389/fonc.2021.562378)

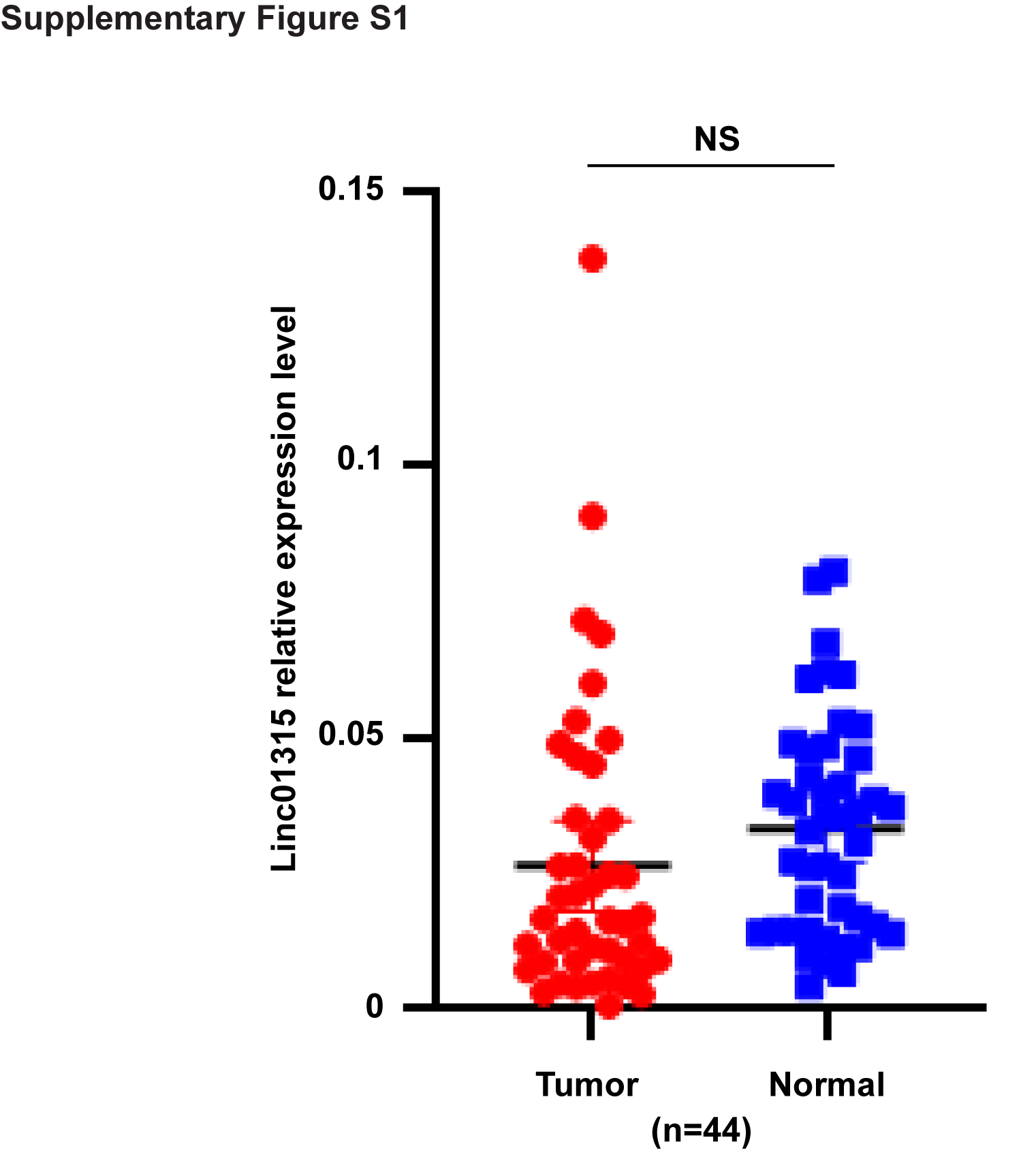

Supplement: Supplementary file 1 [file Image_1.tif]

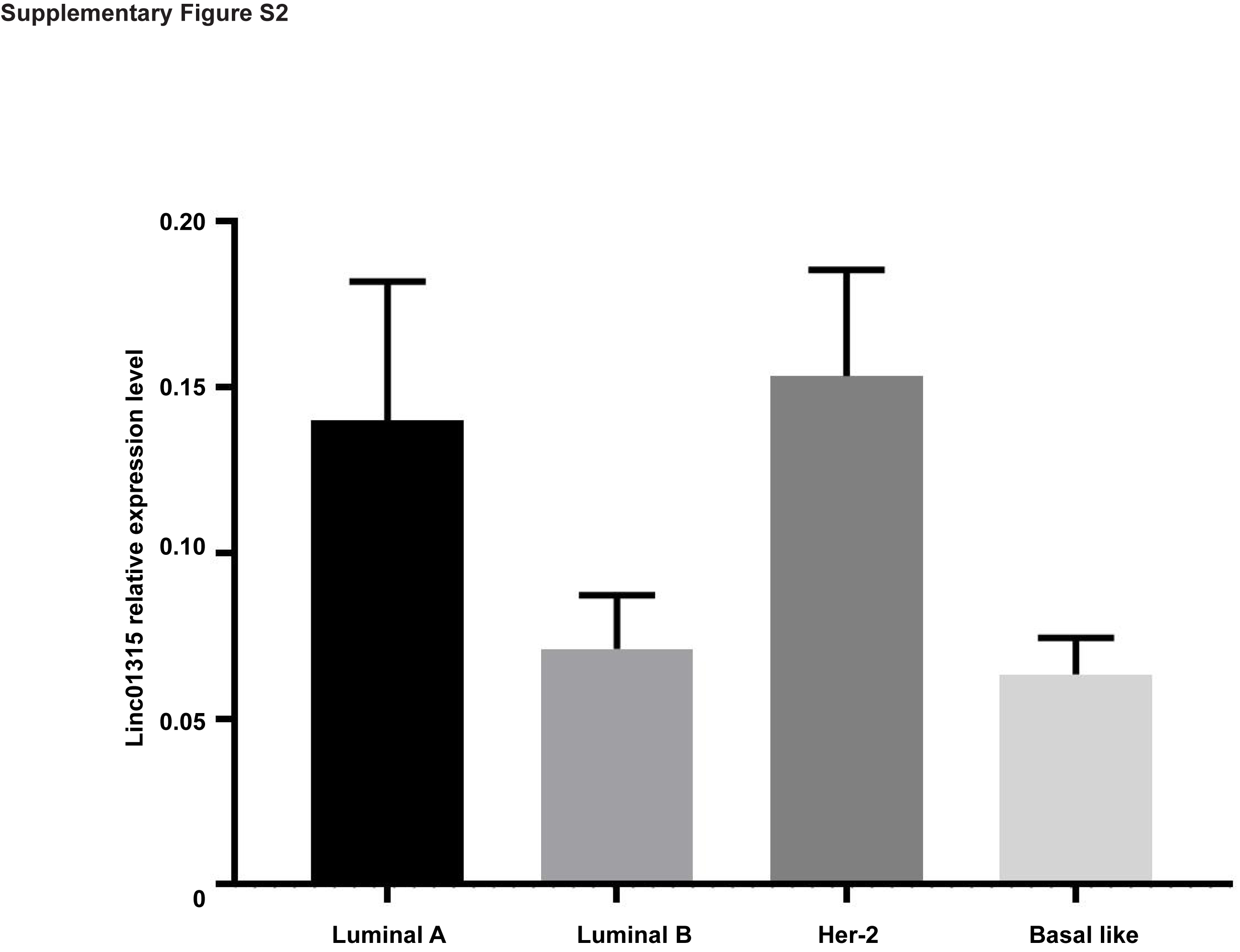

Supplement: Supplementary file 2 [file Image_2.tif]

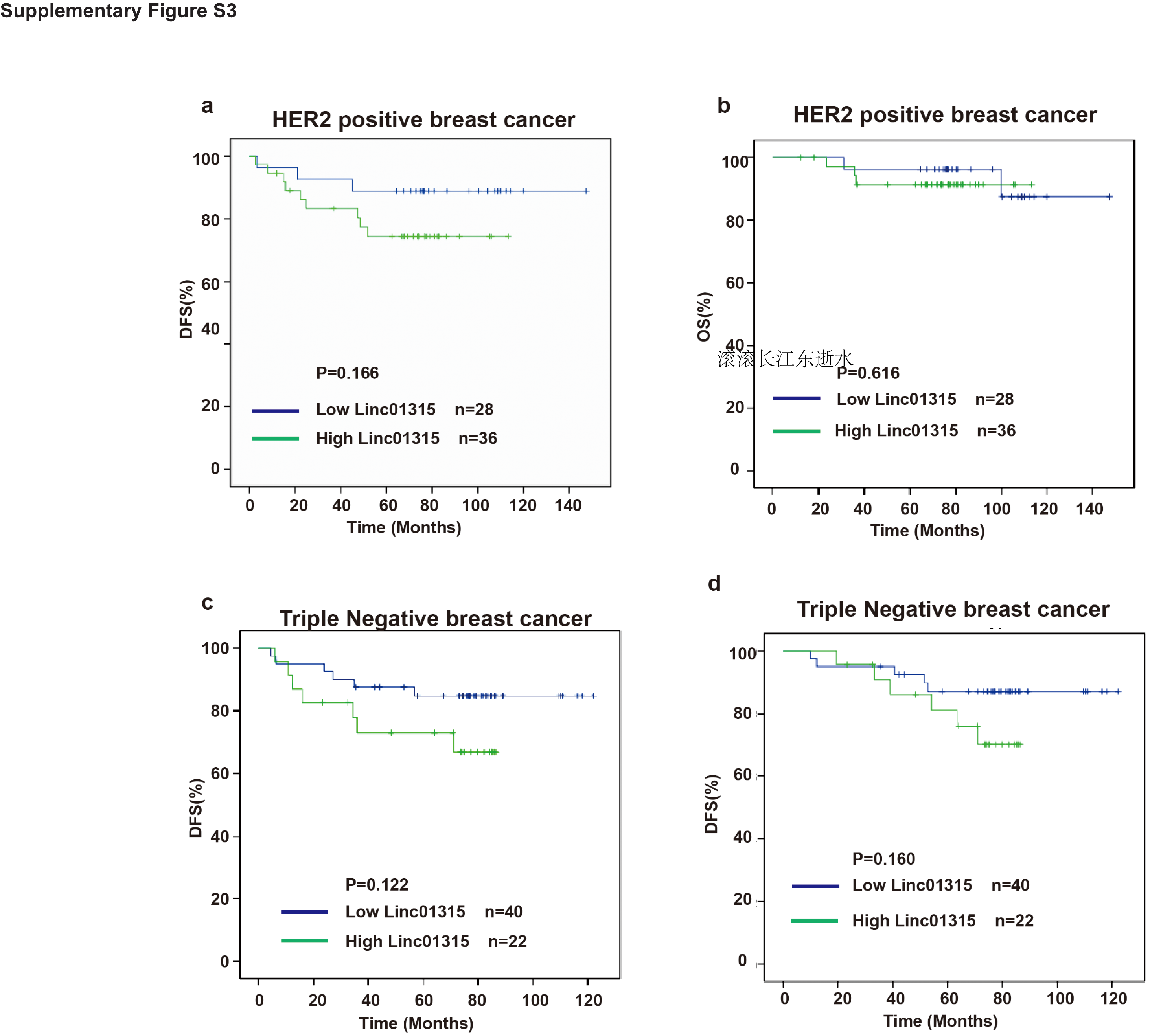

Supplement: Supplementary file 3 [file Image_3.tif]
